# Risk factors for methamphetamine use in youth: a systematic review

**DOI:** 10.1186/1471-2431-8-48

**Published:** 2008-10-28

**Authors:** Kelly Russell, Donna M Dryden, Yuanyuan Liang, Carol Friesen, Kathleen O'Gorman, Tamara Durec, T Cameron Wild, Terry P Klassen

**Affiliations:** 1 Department of Pediatrics, University of Alberta, Edmonton, Canada; 2 School of Public Health, University of Alberta, Edmonton, Canada; 3 Department of Pediatrics, University of Alberta, Aberhart Centre, Room 8213, 11408 University Avenue, Edmonton, Alberta, T6G 2J3, CANADA

## Abstract

**Background:**

Methamphetamine (MA) is a potent stimulant that is readily available. Its effects are similar to cocaine, but the drug has a profile associated with increased acute and chronic toxicities. The objective of this systematic review was to identify and synthesize literature on risk factors that are associated with MA use among youth.

More than 40 electronic databases, websites, and key journals/meeting abstracts were searched. We included studies that compared children and adolescents (≤ 18 years) who used MA to those who did not. One reviewer extracted the data and a second checked for completeness and accuracy. For discrete risk factors, odds ratios (OR) were calculated and when appropriate, a pooled OR with 95% confidence intervals (95% CI) was calculated. For continuous risk factors, mean difference and 95% CI were calculated and when appropriate, a weighted mean difference (WMD) and 95% CI was calculated. Results were presented separately by comparison group: low-risk (no previous drug abuse) and high-risk children (reported previous drug abuse or were recruited from a juvenile detention center).

**Results:**

Twelve studies were included. Among low-risk youth, factors associated with MA use were: history of heroin/opiate use (OR = 29.3; 95% CI: 9.8–87.8), family history of drug use (OR = 4.7; 95% CI: 2.8–7.9), risky sexual behavior (OR = 2.79; 95% CI: 2.25, 3.46) and some psychiatric disorders. History of alcohol use and smoking were also significantly associated with MA use. Among high-risk youth, factors associated with MA use were: family history of crime (OR = 2.0; 95% CI: 1.2–3.3), family history of drug use (OR = 4.7; 95% CI: 2.8–7.9), family history of alcohol abuse (OR = 3.2; 95% CI: 1.8–5.6), and psychiatric treatment (OR = 6.8; 95% CI: 3.6–12.9). Female sex was also significantly associated with MA use.

**Conclusion:**

Among low-risk youth, a history of engaging in a variety of risky behaviors was significantly associated with MA use. A history of a psychiatric disorder was a risk factor for MA for both low- and high-risk youth. Family environment was also associated with MA use. Many of the included studies were cross-sectional making it difficult to assess causation. Future research should utilize prospective study designs so that temporal relationships between risk factors and MA use can be established.

## Background

Methamphetamine (MA), also known as crystal meth, is a synthetic stimulant that affects the brain and central nervous system[1-4]. Smoking is the most common route of administration for MA[5]. When smoked or injected, it produces an initial rush that lasts only a couple of minutes but is intensely pleasurable[5]. This is followed by a prolonged high that results in an extended period of euphoria[5]. The half-life of MA ranges from 10–30 hours depending on the purity of the drug, urine pH, and the amount consumed[2]. Like other psychoactive drugs of abuse, chronic MA use can result in tolerance, where increased amounts of MA are required to produce the same high[6].

Because MA is a stimulant, it produces physiological and psychological effects similar to those elicited by cocaine[1]. MA stimulates the release of dopamine, norepinephrine, and serotonin, and blocks their reuptake[7]. This excess amount of neurotransmitters in the synapses produces sensations of euphoria, lowered inhibitions, feelings of invincibility, increased wakefulness, heightened sexual experiences, and hyperactivity resulting from increased energy for extended periods of time[8]. Deleterious short-term effects include increased heart and respiration rates, hyperthermia, chest pain, hypertension, increased respiration, decreased appetite, anorexia, irritability, confusion, tremors, convulsions, anxiety, aggressiveness, and symptoms of psychosis such as hallucinations and paranoia[4,9,10]. This is followed by mental and physical exhaustion, headaches, irritability, reduced concentration, hunger, decreased energy, anhedonia, and a craving for more MA[3,11]. Cognitive impairments and changes in the brain that result in symptoms similar to those of Parkinson's disease can occur[12,13]. Long-term use of MA use is associated with neurotoxicity, neurodegeneration, and clinical depression that may lead to homicidal and suicidal ideation and action[5].

MA is produced, or "cooked" quickly, reasonably simply, and cheaply by using legal and readily available ingredients, including ephedrine, pseudoephedrine, red phosphorous, iodine, ammonia, paint thinner, lye, camping fuel, drain cleaner, and lithium[5]. These components and cooking tools can be purchased at local drug stores and hardware stores, and recipes can be found on the Internet[14]. Many of the chemicals used in the production of MA are explosive and the generated waste products are corrosive and toxic[15].

In 2004, the US National Survey on Drug Use and Health surveyed persons over the age of 11 and found that 1.4 million people (0.6% of the population) had used MA in the past 12 months, and 600,000 (0.2%) had used it in the previous month[16]. School-based drug surveys administered in Ontario and Manitoba specifically asked about MA use[17,18]. They found that between 2.7% and 3.3% of students reported using MA within the last year. In another Canadian province, 4% (108,000) of Albertans aged 15 or older reported using more than one amphetamine-type stimulant[4]. In 2002, street youth aged 14–30 years were surveyed and 71% of respondents reported using amphetamine-type stimulants and 57% had used them on more than ten occasions[4].

Because MA is easily accessible, relatively cheap, and has reinforcing properties, chronic use can pose a significant danger[4]. If risk factors for MA use could be identified, physicians and other health care professionals who work with youth may be better equipped to identify MA users and develop education and prevention programs that could be targeted to youth at greater risk for using MA. Thus, we performed a systematic review to identify factors at the individual, family, and community level that are associated with MA use among children and adolescents.

## Methods

### Literature search

We searched over 40 electronic databases, including MEDLINE^®^, Ovid MEDLINE^® ^In-Process & Other Non-Indexed Citations, EBM Reviews – Cochrane Central Register of Controlled Trials, EMBASE, CINAHL^®^, PsycINFO^®^, International Pharmaceutical Abstracts, Pascal, Global Health, Science Citation Index Expanded and Social Sciences Citation Index (via Web of Science^®^), Social Sciences Abstracts, and Psychology and Behavioral Sciences Collection. Trials registers (Current Controlled Trials, ClinicalTrials.gov, the Australian Clinical Trials Registry, and the National Research Register in the United Kingdom) were searched for additional trials. Search terms such as methamphetamine, variant spellings of methamphetamine, amphetamine-related disorders, and crystal meth, were adapted for each database and appropriate subject headings and keywords were used. In addition, an extensive search for grey literature was conducted. Hand searching was conducted in relevant scientific journals, scientific meetings, and the reference lists of relevant reviews and included studies were reviewed. We restricted the search results to English-language studies. The literature search is considered up to date as of May 15, 2006. Full search strategies and lists of resources searched are available [see Additional file 1].

### Study selection and inclusion criteria

Two reviewers independently screened the titles and, when available, the abstracts. Based on general inclusion criteria, studies were classified as "potentially relevant", "irrelevant", and "unclear". The full text of studies described as "potentially relevant" and "unclear" was obtained and two reviewers independently applied the specific inclusion criteria. Studies were included if they compared children ≤ 18 years of age who did and did not use MA (the comparison group could be other drug users or children who do not use drugs). The following study designs were included: case-control, cohort, and cross-sectional. Studies were excluded if they did not have a comparison group, if the outcomes were not measured quantitatively or if they were uncontrolled before and after studies, case-series, or case studies. Disagreements were resolved through discussion or through third party adjudication, as necessary.

### Methodological quality assessment

Two reviewers independently assessed methodological quality; discrepancies were resolved through consensus or by third party adjudication as required. Observational studies were assessed using the Downs and Black checklist[19]. This tool comprises six sections that assess reporting, external validity, internal validity (bias), internal validity (confounding), and power.

### Data extraction

Data were extracted by one reviewer and checked for accuracy and completeness by a second reviewer. A standard data extraction form was developed and the data were subsequently entered into an electronic database. For each included study, we extracted information about the population (demographics and sources), type of study (study design, prospective or retrospective data collection), definitions and details of risk factors, and the numeric results.

### Data analysis

After reviewing the studies that met our inclusion criteria, we made a post hoc decision to group the studies and conduct the data analysis by the nature of the comparison group: 1) youth who did not use illicit drugs (referred to as "low-risk") and 2) youth who abused illicit drugs other than MA or were recruited from juvenile detention centers (referred to as "high-risk"). For dichotomous risk factors (e.g., sex) we calculated pooled odds ratios (OR) with 95% confidence intervals (95% CI), where appropriate. For continuous risk factors (e.g., years of education) we calculated weighted mean difference (WMD) and 95% CI using the DerSimonian and Laird random effects model[20]. A random effects model was used because it allows for combining heterogeneous results where the heterogeneity cannot readily be explained[21]. The resulting estimate is more conservative because the resulting confidence intervals are wider. Statistical heterogeneity was assessed using the I^2 ^statistic, which describes the percentage of total variation across studies that is due to heterogeneity rather than chance. For this review a value greater than 50% was considered as substantial heterogeneity[22]. In instances where only one study reported the specific risk factor or where pooling was not appropriate, the effect estimate (OR or WMD) and 95% CI were reported for each risk factor.

## Results

### Literature search

**Figure 1:**
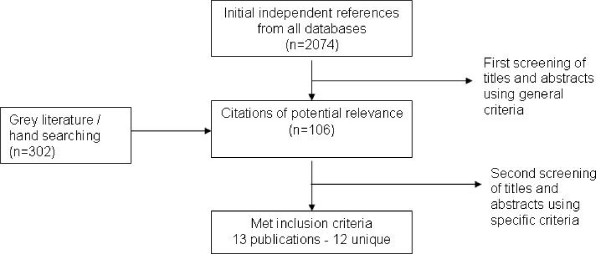
Study retrieval and selection of studies investigating risk factors for MA use.

The database, grey literature searches and hand searching yielded 2,376 potentially relevant studies. In total, 106 unique studies were reviewed and 13 met our inclusion criteria. Of these, two studies assessed risk factors in the same population and are treated as one study for the purposes of this report[23]. Therefore, our review includes 13 publications but only 12 unique studies. Study retrieval and selection is outlined in Figure 1.

Studies were excluded from the review for the following reasons: not relevant to the topic (n = 34), incorrect study population (n = 33), inappropriate study design (n = 12), not primary research (n = 13), and inadequate data (n = 1).

### Description of included studies

The characteristics of the 12 included studies and populations are presented in Tables 1 [see Additional file 2] and 2 [see Additional file 3]. Most studies relied on self-reported MA use. Three studies administered urine tests to determine MA use[24-26] and one study diagnosed children with MA dependence[27]. The remaining studies relied on self-reported MA use. Seven studies were conducted in North America [27-33] and the remaining five in Asia[23,24,26,34,35]. The majority of the studies were published recently; the median year of publication was 2004. The median sample size was 604 and ranged from 60 to 78,715. Three studies used a case-control design and the remaining nine were cross-sectional.

**Table 1:** Risk factors for MA: quality of included studies

**Study Year**	**Study Design**	**Downs and Black Score**	**Present Adjusted OR**	**Funding Source**
**Low-risk youth as the comparison group**				

Lampinen 2006	Cross-sectional	15	Yes	Other

Oetting 2000	Cross-sectional	12	No	Government

Sattah 2002	Cross-sectional	15	Yes	Government

Yen 2006^a^	Case-control	15	Yes	Government

Yen 2004^b^	Cross-sectional	13	No	NR

**High-risk youth as the comparison group**				

Kim 2002	Cross-sectional	16	No	Government

Miura 2006	Case-control	15	Yes	NR

Palmer 2005	Case-control	13	No	NR

Rawson 2005	Cross-sectional	14	Yes	NR

Shillington 2005	Cross-sectional	18	Yes	Other

Shillington 2003	Cross-sectional	17	No	NR

Uchida 1995	Cross-sectional	12	No	NR

^a ^Age and sex matched

^b ^Sex matched

**Table 2:** Comparing MA users to Low-Risk Youth

**Risk Factor**	**Study**	**Statistical Measure**	**Point Estimate (95% CI)**	**Conclusion**
Sex^a^	Oetting 2000	OR	0.72 (0.70, 0.75)	Odds of using MA were higher for males.
	Sattah 2002	OR	0.34 (0.27, 0.43)	
Years of education	Yen 2004	MD	2.70 (2.36, 3.04)	Odds of using MA were higher for those with less education.
	Yen 2006	MD	2.60 (2.40, 2.80)	
	Pooled^b^	WMD	2.63 (2.45, 2.80)	
	Sattah 2002	OR	1.31 (1.06, 1.62)	
Sexual behavior	Sattah 2002	OR	2.79 (2.25, 3.46)	Odds of using MA was higher for those who had previously engaged in sexual intercourse.
	Yen 2004	OR	31.79 (15.56, 64.93)	
Alcohol use	Sattah 2002	OR	8.02 (4.53, 14.18)	Odds of using MA was higher for those who drink alcohol.
	Yen 2006	OR	51.31 (12.27, 214.68)	
Heroin/opiate use	Sattah 2002	OR	30.66 (9.38, 100.17)	Odds of using MA was higher for those who had a history of heroin/opiate use.
	Yen 2006	OR	22.53 (1.24, 409.59)	
Smoking	Sattah 2002	OR	13.72 (10.69, 17.60)	Odds of using MA were higher for those who smoke.
	Yen 2006	OR	154.85 (81.95, 292.60)	
Family history of drug use	Yen 2006	OR	8.65 (3.88, 19.25)	Odds of using MA were higher for youth with family history of drug use.
Homosexual or bisexual	Lampinen 2006	OR	17.02 (4.83, 60.01)	Odds of using MA were higher for youth who were homosexual or bisexual.
Experiencing disruptive parenting	Yen 2006	OR	7.84 (5.25, 11.71)	Odds of using MA were higher for youth who experienced disruptive parenting.
Peers using or providing MA	Yen 2006	OR	40.94 (24.64, 68.03)	Odds of using MA were higher for youth with peers using or providing MA.
Engaging in unprotected sex	Yen 2004	OR	15.68 (8.04, 30.58)	Odds of using MA were higher for youth who engaged in unprotected sex.
Engaging in unplanned sex under the influence of alcohol	Yen 2004	OR	70.42 (9.34, 531.06)	Odds of using MA were higher for youth who engaged in unplanned sex under the influence of alcohol.
Engaging in sex with an alcohol-intoxicated partner	Yen 2004	OR	29.33 (6.70, 128.36)	Odds of using MA were higher for youth who engaged in sexual intercourse with an alcohol-intoxicated partner.
Any psychiatric disorder	Yen 2006	OR	3.05 (2.12, 4.39)	Odds of using MA were higher for youth who had any psychiatric disorder.
Adjustment disorder	Yen 2006	OR	2.89 (1.53, 5.47)	Odds of using MA were higher for youth who had adjustment disorder.
Conduct disorder	Yen 2006	OR	31.91 (16.06, 63.41)	Odds of using MA were higher for youth who had conduct disorder.
Attention-deficit hyperactivity disorder	Yen 2006	OR	2.84 (1.81, 4.47)	Odds of using MA were higher for youth who had ADHD.

^a ^Female = 1, Male = 0

^b ^Combine Yen 2004 and Yen 2006, I^2 ^= 0%

In five studies, youth who reported using MA were compared to youth who did not use illicit drugs, hereafter referred to as "low-risk" youth. These low-risk youth were sampled from school populations. For the remaining seven studies, youth who reported using MA were compared to youth who abused illicit drugs other than MA, and/or youth who were sampled from juvenile detention centers, hereafter referred to as "high-risk" youth. Youth were recruited from a variety of locations, including schools, detention centers, juvenile homes, or treatment facilities. One study included youth up to the age of 24 and one study did not report the age of participants. The studies assessed a variety of risk factors, including demographic variables, mental health status, and risky behaviors.

### Methodological quality of included studies

The median Downs and Black score was 15 of a possible score of 29 and ranged from 12 to 28 [Table 1] suggesting a risk of bias for those studies that received lower quality scores. Six of the twelve studies reported adjusted results, that is, the risk factor of interest was adjusted for other potentially confounding risk factors[23,26,28,31,32,34]. Six studies disclosed their funding source and the most common source was a government agency[23,26,29,30,32].

### Quantitative results: comparing MA users to low-risk youth

Five studies compared risk factors for MA among low-risk youth, as defined above (Table 2) [23,24,26,28,29]. There was considerable heterogeneity among the studies, which precluded the calculation of pooled estimates of effects in most cases. Pooled estimates are presented where appropriate.

#### Sex

Two cross-sectional studies examined sex as a risk factor and both independently indicated a significant association showing that males are more likely to use MA than females[26,29].

#### Ethnicity

One cross-sectional study using survey data examined ethnicity as a risk factor for MA[29]. The results showed that Caucasian youth were more likely to use MA than African-American youth and Asian youth. However, Caucasian youth were significantly less likely to use MA than Hispanic and Native American youth.

#### Years of education

One case-control[23] and two cross-sectional studies[24,26] examined education as a risk factor for MA use and all three studies concluded that MA use was significantly associated with fewer years of education. Two studies reported the mean years of education among MA users and non-MA users. The pooled WMD indicated that non-MA users had more years of education than youth who used MA (WMD = 2.63; 95% CI: 2.45 to 2.80)[24,25]. The third study categorized respondents' educational attainment of Grades 1–3 versus Grades 4 or more and also showed that non-MA users were 1.3 times more likely to have more education (95% CI: 1.06 to 1.62)[26].

#### Sexual behavior

Two cross-sectional studies independently reported a significant association between having ever previously engaged in sexual intercourse and using MA[8,36]. One study found the following behaviors were significantly associated with MA use: engaging in unprotected sex, engaging in unplanned sex under the influence of alcohol, and engaging in sexual intercourse with an alcohol-intoxicated partner[24].

#### Alcohol, cigarette and opiate use

Two studies (one case-control[23] and one cross-sectional[26]) independently reported statistically significant associations between alcohol use, smoking, and heroin/opiate use.

#### Psychiatric disorders

Two studies of the same population examined the relationship between psychiatric disorders and MA use; however, the studies did not examine the same psychiatric conditions and their results could not be pooled[23,25]. The presence of the following conditions were found to be significantly associated with MA use: having any psychiatric disorder, adjustment disorder, conduct disorder, and attention deficit and hyperactivity disorder (ADHD). Oppositional defiance disorder, anxiety disorder, major depressive disorder, dysthmic disorder, bipolar disorder, and eating disorder were not significantly associated with MA use among low-risk youth.

#### Other risk factors

Several other risk factors were associated with MA use among low-risk youth: being homosexual or bisexual[28], experiencing disruptive parenting[23], peers using or providing MA[23], and family history of drug use[23].

#### Multivariate analyses

Three studies conducted adjusted or multivariable logistic regression analyses (i.e., the risk factor of interest was adjusted for other potentially confounding factors). Lampinen et al. found that age and sexual preference were significant risk factors for MA use; sex was not found to be a risk factor in their analysis[28]. After adjusting for other psychiatric conditions and peer and family characteristics, Sattah et al. reported that the following risk factors were associated with MA use: recent alcohol or tobacco use, history of marijuana use, not having a family confidant, peer pressure, having a positive attitude towards MA use, and sexual experience[26]. The third study found that conduct disorder, a positive attitude toward MA use, poor understanding of MA use, disruptive parenting, low level of caregiver education, friends using or providing MA, and a more interactive interaction with peers were significant risk factors for MA[25]. Sex was not found to be statistically significant in this multivariable analysis.

### Quantitative results: comparing MA users to high-risk youth

Seven studies examined risk factors for MA among high-risk youth (Table 3)[27,30-35]. Because of substantial heterogeneity among the studies, pooling of estimates was generally not appropriate.

**Table 3:** Comparing MA Users to High-Risk Youth

**Risk Factor**	**Study**	**OR (95% CI)**	**Conclusion**
Sex^a^	Uchida 1995	6.55 (2.34, 18.34)	Females were more likely to use MA than males.
	
	Kim 2002	1.53 (1.27, 1.85)	
	
	Shilungton 2003	4.00 (3.49, 4.58)	
	
	Rawson 2005	9.53 (5.40, 16.79)	
	
	Miura 2006	4.57 (2.92, 7.17)	

Age^b^	Rawson 2005	2.10 (0.84, 5.26)	Age was no significantly associated with MA use.

Alcohol use	Rawson 2005	1.04 (0.56, 1.95)	No association between a history of alcohol use and MA use

Family history of crime^c^	Miura 2006	2.00 (1.22, 3.29)	Odds of using MA was higher for youth with family history of crime.

Family history of drug use	Miura 2006	4.70 (2.79, 7.90)	Odds of using MA was higher for youth with family history of drug use.

Family history of alcohol abuse	Uchida 1995	3.61 (1.39, 9.39)	Odds of using MA was higher for youth with family history of alcohol abuse.
		
	Miura 2006	2.94 (1.44, 6.00)	
		
	Pooled^c^	3.16 (1.78, 5.61)	

Child abuse	Uchida 1995	3.13 (1.24, 7.92)	Odds of using MA was higher for youth who experienced child abuse; the association was not statistically significant.
		
	Miura 2006	1.49 (0.73, 3.07)	
		
	Pooled^d^	2.04 (0.99, 4.17)	

Receiving psychiatric treatment	Miura 2006	6.78 (3.55, 12.94)	Odds of using MA was higher for youth who were receiving psychiatric treatment.

Greater than two admissions to juvenile home	Miura 2006	2.70 (1.77, 4.13)	Odds of using MA was higher for youth with greater than two admissions to juvenile home.

History of violence	Miura 2006	0.35 (0.20, 0.62)	Odds of using MA was lower for youth with history of violence.

Strict parental monitoring	Shillington 2005	0.25 (0.11, 0.57)	Odds of using MA was lower for youth with strict parental monitoring.

^a ^Female = 1, Male = 0

^b ^13–14 years vs. 15–18 years

^c ^I^2 ^= 0%

^d ^I^2 ^= 34.7%

#### Sex

Five studies (one case-control and four cross-sectional) independently reported that female sex was significantly associated with MA use[30,31,33-35].

#### Age

One cross-sectional study categorized participants into two age groups: 13–14 years and 15–18 years[31]. The results show that age was not significantly associated with MA use.

#### Ethnicity

Three studies (one case-control and two cross-sectional) examined the association between ethnicity and MA use among high-risk youth[27,31,32]. Compared to African-American and Asian youth, Caucasian youth were significantly more likely to use MA. There was no significant difference in MA use between Caucasian versus Hispanic, Asian, or Native American.

#### Alcohol use

One cross-sectional study found no association between a history of alcohol use and MA use[31].

#### Family history

One cross-sectional study found that a family history of crime or drug use was significantly associated with MA use[34]. The pooled analysis of two cross-sectional studies showed a significant association between family history of alcohol abuse and MA use[34,35].

#### Child abuse

The pooled analysis of one cross-sectional and one case-control study showed a two-fold association between children who experienced abuse and MA use; however, the result was not statistically significant[34,35].

#### Other risk factors

The following factors were significantly associated with MA use: receiving psychiatric treatment, greater than two admissions to juvenile home, and history of violence[34]. However, strict parental monitoring was found to be protective for MA use among high-risk youth[32].

#### Multivariable analyses

Three studies conducted a multivariable logistic regression analysis. Shillington et al. found that strict parental monitoring, after controlling for age, was a statistically significant protective factor against MA use among high-risk youth[32]. After controlling for age and race, Rawson et al. found that female sex was significantly associated with MA use[31]. In the third study, female sex, age, more than two admissions to a juvenile home, non-violent history, psychiatric treatment, family history of drug misuse, and child abuse were significantly associated with MA use; a family history of crime was not significantly associated with MA use[34].

## Discussion and conclusion

This systematic review presents the best-available evidence regarding risk factors for MA use among youth. An exhaustive search of over 40 electronic databases, grey literature, and hand searching identified 12 unique studies that met our inclusion criteria. The majority of the studies (9/12) were cross-sectional in design and therefore it is not possible to determine whether the risk factors precede or follow MA use.

Because we believe that factors associated with MA use may differ among socially integrated (i.e., low-risk) and marginalized (i.e., high-risk) youth, we chose to analyze these studies separately. Compared to low-risk youth, there were some clear patterns of risk factors associated with MA use. A history of engaging in a variety of risky behaviors (e.g., sexual activity [planned, unplanned, or under the influence of alcohol], alcohol consumption, and opiate use) was significantly associated with MA use among low-risk youth. Engaging in high-risk behavior may be a gateway for MA use or vice versa. Homosexual or bisexual lifestyle is also a risk factor. This is not surprising, as MA is believed to heighten sexual pleasure and gay and bisexual men cite this as a reason for using MA[36]. A history of a psychiatric disorder and, in particular, adjustment disorder, conduct disorder, or ADHD, is a risk factor for MA use. This is consistent with previous research that shows psychiatric conditions to be risk factors for drug use in general[37].

Several risk factors were associated with MA use among high risk youth (i.e., those that used other illicit drugs or were in detention/juvenile centers). Unlike low-risk youth, females were significantly more likely to use MA. Youth who grew up in an unstable family environment (e.g., family history of crime, alcohol use, and drug use) were significantly more likely to use MA. While child abuse was not a significant risk factor, it approached statistical significance. High-risk youth who had received treatment for psychiatric conditions were more likely to use MA. One study found that strict parental monitoring was found to be protective against MA use among this group of youth.

### Limitations

There were only 12 studies that met our inclusion criteria. These studies were fairly heterogeneous, which precluded pooling of results for most risk factors. Furthermore, many of the risk factors were assessed in only one study and the sample size was small. This is reflected in the wide confidence intervals and imprecise effect estimates. Most studies were either cross-sectional or retrospective making it impossible to assess a causal relationship between the risk factors and MA use. As with any systematic review, there is the possibility of publication and selection bias. However, we feel the risk for publication bias was minimized by our exhaustive search process. In addition to electronic databases, the reference lists of the included studies were searched, relevant conference proceedings and key journals were hand searched, and a thorough grey literature search was conducted.

### Implications for clinicians and front-line workers

Youth who engage in risk-taking behaviors, live in an unstable home environment, have a psychiatric condition, and have peers that use or sell MA have a higher propensity for MA use. In order to identify youth at risk for MA use, health care workers and counsellors need to conduct a holistic assessment that includes psychiatric, lifestyle, and family history.

## Authors' contributions

KR co-ordinated the project, prepared the manuscript, and assisted with assessing studies for inclusion, methodological quality assessment, data extraction, summarizing the qualitative results.

DD provided methodological expertise and provided feedback on the manuscript.

YL completed the statistical analysis and provided feedback on the manuscript.

CF designed and executed the literature searches. She co-ordinated and assisted with all stages of the systematic review process, including manuscript preparation, related to the policy component.

KO assisted with assessing studies for inclusion, methodological quality assessment, data extraction, summarizing the qualitative results, and provided feedback on the manuscript.

TD assisted with designing and executing the literature searches and provided feedback on the manuscript. She also assisted with assessing studies for inclusion, methodological quality assessment, and data extraction.

TCW provided methodological and clinical expertise and provided feedback on the manuscript.

TPK provided methodological and clinical expertise and provided feedback on the manuscript.

## Pre-publication history

The pre-publication history for this paper can be accessed here:



## Supplementary Material

Additional File 1 **Electronic Databases and Search Strategies**. This file contains the literature databases and search strategies. Click here for file

Additional File 2 **Table**1. **Risk factors for methamphetamine use: description of the population.** This file contains a table. Click here for file

Additional File 3 **Table**2. **Risk factors for methamphetamine use: description of the risk factors.** The file contains a table. Click here for file
