# Supplementary material for: Risk factors for methamphetamine use in youth: a systematic review
Source: BMC Pediatr. 2008 Oct 28;8:48. doi: 10.1186/1471-2431-8-48 (PMC2588572; doi:10.1186/1471-2431-8-48)
Supplement: Additional File 1 — Electronic Databases and Search Strategies. This file contains the literature databases and search strategies. [file 1471-2431-8-48-S1.doc]

**Electronic Databases**

| **Electronic Databases Searched** | **Search Strategies** |
| --- | --- |
| **MEDLINE®**  Ovid version rel10.3.0  1966 to May Week 3 2006  Searched May 24, 2006 | 1. exp Amphetamine-Related Disorders/  2. exp Methamphetamine/  3. (methamphetamine$ or metamphetamine$).mp.  4. 537-46-2.rn.  5. or/1-4  6. RANDOMIZED CONTROLLED TRIAL.pt.  7. CONTROLLED CLINICAL TRIAL.pt.  8. RANDOMIZED CONTROLLED TRIALS/  9. RANDOM ALLOCATION/  10. DOUBLE BLIND METHOD/  11. SINGLE-BLIND METHOD/  12. CLINICAL TRIAL.pt.  13. exp CLINICAL TRIALS/  14. ((clin$ or control$) adj25 trial$).ti,ab.  15. ((singl$ or doubl$ or trebl$ or tripl$) adj25 (blind$ or mask$)).ti,ab.  16. PLACEBOS/  17. placebo$.ti,ab.  18. random$.ti,ab.  19. RESEARCH DESIGN/  20. exp case-control studies/ or exp retrospective studies/  21. exp cohort studies/ or exp longitudinal studies/ or exp follow-up studies/ or exp prospective studies/ or comparative study/ or exp Evaluation Studies/  22. exp Cross-Sectional Studies/  23. (cohort or control$ or observational or prospectiv$ or "time series" or "time-series" or "case comparison" or "case-comparison" or "case referent" or "case-referent" or "cross sectional" or "cross-sectional" or risk$ or effectiveness or "multi center" or "multi centre" or multicenter or multicentre or multisite or "multi site" or "before and after").ti,ab.  24. exp treatment outcome/  25. exp Feasibility Studies/  26. exp Multicenter Studies/  27. multicenter study.pt.  28. exp Patient Compliance/  29. exp Health Promotion/  30. (pc or th or rh or sd or lj).fs.  31. exp Risk Factors/  32. predict$.ti,ab.  33. exp Causality/  34. exp Primary Prevention/  35. prevent$.ti,ab.  36. exp Behavior Therapy/  37. exp Substance Abuse Treatment Centers/  38. or/6-37  39. ANIMALS/ not HUMANS/  40. 38 not 39  41. 5 and 40  42. limit 41 to ("all infant (birth to 23 months)" or "all child (0 to 18 years)" or "preschool child (2 to 5 years)" or "child (6 to 12 years)" or "adolescent (13 to 18 years)")  43. exp Public Policy/  44. exp Legislation/  45. "drug and narcotic control".sh.  46. exp Law Enforcement/  47. exp Criminal Law/  48. exp Police/  49. (regulation$ or arrest$).ti,ab.  50. (policy or policies).ti,ab.  51. or/43-50  52. 5 and 40 and 51  53. 42 or 52  54. limit 53 to english language  55. or/6-19  56. 5 and 55  57. 56 not 39  58. limit 57 to english language  Please note: Lines 1-54 were run initially for all of the questions; Lines 55-58 were run to look for randomized controlled trials related to the treatment of methamphetamine use in adults. |
| **Ovid MEDLINE® In-Process & Other Non-Indexed Citations**  Ovid version rel10.3.0 May 30, 2006  Searched May 30, 2006 | 1. methamphetamine$.mp.  2. methylamphetamine$.mp.  3. or/1-2  4. "randomi?ed controlled trial$".mp.  5. "controlled clinical trial$".mp.  6. "random allocation".mp.  7. ("double blind" adj3 method$).mp.  8. ("single blind" adj3 method$).mp.  9. ((clin$ or control$) adj25 trial$).mp.  10. ((singl$ or doubl$ or trebl$ or tripl$) adj25 (blind$ or mask$)).mp.  11. placebo$.mp.  12. random$.mp.  13. "research design$".mp.  14. "clinical research".mp.  15. (cohort or control$ or observational or prospectiv$ or "time series" or "time-series" or "case comparison" or "case-comparison" or "case referent" or "case-referent" or "cross sectional" or "cross-sectional" or risk$ or effectiveness or "multi center" or "multi centre" or multicenter or multicentre or multisite or "multi site" or "before and after" or predict$ or prevent$).ti,ab.  16. or/4-15  17. 3 and 16  18. (child$ or schoolchild$ or paediatric$ or pediatric$ or infan$ or adolescen$ or juvenil$ or teen or teens or teenage$ or youth or youths or highschool$ or "high school$").mp.  19. 17 and 18  20. (regulation$ or arrest$ or legislation or policy or policies or legislative or production or supply or trafficking or ((lab$ or laborator$) adj2 (closure$ or seizure$)) or possession or law$ or enforcement).ti,ab.  21. 17 and 20  22. 19 or 21  23. or/4-12  24. 3 and 23  Please note: Lines 1-22 were run initially for all of the questions; Lines 23-24 were run to look for randomized controlled trials related to the treatment of methamphetamine use in adults. |
| **EBM Reviews - Cochrane Central Register of Controlled Trials**  Ovid version rel10.3.0  2nd Quarter 2006  Searched May 29, 2006 | 1. exp Amphetamine-Related Disorders/  2. exp Methamphetamine/  3. (methamphetamine$ or metamphetamine$ or methylamphetamine$).mp.  4. or/1-3  5. RANDOMIZED CONTROLLED TRIAL.pt.  6. CONTROLLED CLINICAL TRIAL.pt.  7. RANDOMIZED CONTROLLED TRIALS/  8. RANDOM ALLOCATION/  9. DOUBLE BLIND METHOD/  10. SINGLE-BLIND METHOD/  11. CLINICAL TRIAL.pt.  12. exp CLINICAL TRIALS/  13. ((clin$ or control$) adj25 trial$).ti,ab.  14. ((singl$ or doubl$ or trebl$ or tripl$) adj25 (blind$ or mask$)).ti,ab.  15. PLACEBOS/  16. placebo$.ti,ab.  17. random$.ti,ab.  18. RESEARCH DESIGN/  19. exp case-control studies/ or exp retrospective studies/  20. exp cohort studies/ or exp longitudinal studies/ or exp follow-up studies/ or exp prospective studies/ or comparative study/ or exp Evaluation Studies/  21. exp Cross-Sectional Studies/  22. (cohort or control$ or observational or prospectiv$ or "time series" or "time-series" or "case comparison" or "case-comparison" or "case referent" or "case-referent" or "cross sectional" or "cross-sectional" or risk$ or effectiveness or "multi center" or "multi centre" or multicenter or multicentre or multisite or "multi site" or "before and after").ti,ab.  23. exp treatment outcome/  24. exp Feasibility Studies/  25. exp Multicenter Studies/  26. multicenter study.pt.  27. exp Patient Compliance/  28. exp Health Promotion/  29. (pc or th or rh or sd or lj).fs.  30. exp Risk Factors/  31. predict$.ti,ab.  32. exp Causality/  33. exp Primary Prevention/  34. prevent$.ti,ab.  35. exp Behavior Therapy/  36. exp Substance Abuse Treatment Centers/  37. or/5-36  38. ANIMALS/ not HUMANS/  39. 37 not 38  40. 4 and 39  41. exp child/  42. exp infant/  43. adolescent/  44. exp pediatrics/  45. (child$ or schoolchild$ or paediatric$ or pediatric$ or infan$ or adolescen$ or juvenil$ or teen or teens or teenage$ or youth or youths or highschool$ or "high school$").mp.  46. or/41-45  47. 40 and 46  48. exp Public Policy/  49. exp Legislation/  50. "drug and narcotic control".sh.  51. exp Law Enforcement/  52. exp Criminal Law/  53. exp Police/  54. (regulation$ or arrest$).ti,ab.  55. (policy or policies or legislative or production or supply or trafficking or ((lab$ or laborator$) adj2 (closure$ or seizure$)) or possession or law$ or enforcement).ti,ab.  56. or/48-55  57. 4 and 39 and 56  58. 47 or 57  59. or/5-18  60. 4 and 59  Please note: Lines 1-58 were run initially for all of the questions; Lines 58-60 were run to look for randomized controlled trials related to the treatment of methamphetamine use in adults. |
| **EMBASE**  Ovid version rel10.3.0  1988 to 2006 Week 21  Searched May 29, 2006 | 1. exp METHAMPHETAMINE/  2. (methamphetamine$ or metamphetamine$ or methylamphetamine$).mp.  3. 537-46-2.rn.  4. or/1-3  5. Randomized Controlled Trial/  6. exp Randomization/  7. Double Blind Procedure/  8. Single Blind Procedure/  9. Clinical Trial/  10. ((clin$ or control$) adj25 trial$).mp.  11. ((singl$ or doubl$ or trebl$ or tripl$) adj25 (blind$ or mask$)).mp.  12. exp Placebo/  13. (placebo$ or random$).mp.  14. exp Methodology/  15. "analytic stud$".mp.  16. exp case-control study/ or exp retrospective study/  17. exp cohort analysis/ or exp longitudinal study/ or exp prospective study/ or exp Comparative Study/ or exp Evaluation/  18. (("follow up" or follow-up) adj stud$).mp.  19. RISK ASSESSMENT/ or exp RISK/  20. (cohort or control$ or observational or prospectiv$ or "time series" or "time-series" or "case comparison" or "case-comparison" or "case referent" or "case-referent" or "cross sectional" or "cross-sectional" or risk$ or effectiveness or "multi center" or "multi centre" or multicenter or multicentre or multisite or "multi site" or "before and after").ti,ab.  21. exp treatment outcome/  22. (predict$ or outcome$).ti,ab.  23. exp public health/  24. exp Outcomes Research/  25. exp Feasibility Study/  26. Multicenter Study/  27. multisite.ti,ab.  28. exp Follow Up/  29. exp Patient Compliance/  30. exp Health Promotion/  31. (pc or th or rh or ct).fs.  32. exp RISK FACTOR/  33. exp Epidemiology/  34. exp primary prevention/  35. prevent$.ti,ab.  36. exp behavior therapy/  37. exp drug dependence treatment/  38. exp Time Series Analysis/  39. Controlled Study/  40. exp treatment planning/  41. exp Health Program/  42. or/5-41  43. Nonhuman/  44. 42 not 43  45. 4 and 44  46. limit 45 to (child or preschool child <1 to 6 years> or school child <7 to 12 years> or adolescent <13 to 17 years>)  47. (adolescen$ or juvenil$ or teen or teens or teenage$ or youth or youths or highschool$ or "high school$").mp.  48. 45 and 47  49. 46 or 48  50. exp policy/  51. exp legal aspect/  52. (drug and narcotic control).mp.  53. exp police/  54. exp health care policy/  55. (arrest$ or policy or policies or legislation$ or legislative or trafficking or law$).ti,ab.  56. or/50-55  57. 45 and 56  58. 49 or 57  59. limit 58 to english language  60. or/5-13  61. 4 and 60  62. 61 not 43  63. limit 62 to english language  Please note: Lines 1-59 were run initially for all of the questions; Lines 60-63 were run to look for randomized controlled trials related to the treatment of methamphetamine use in adults. |
| **CINAHL®**  Ovid version rel10.3.0  1982 to May Week 3 2006  Searched May 29, 2006 | 1. exp METHAMPHETAMINE/  2. methamphetamine$.mp.  3. or/1-2  4. limit 3 to (preschool child <2 to 5 years> or child <6 to 12 years> or adolescence <13 to 18 years>)  5. exp Methamphetamine/lj [Legislation and Jurisprudence]  6. (legislation$ or policy or policies or production or supply or law$ or enforcement).ti,ab.  7. lj.fs.  8. or/6-7  9. 3 and 8  10. 5 or 9  11. 4 or 10  12. limit 11 to english language  13. "randomi?ed controlled trial$".mp.  14. "controlled clinical trial$".mp.  15. "random allocation".mp.  16. ("double blind" adj3 method$).mp.  17. ("single blind" adj3 method$).mp.  18. ((clin$ or control$) adj25 trial$).mp.  19. ((singl$ or doubl$ or trebl$ or tripl$) adj25 (blind$ or mask$)).mp.  20. placebo$.mp.  21. random$.mp.  22. or/13-21  23. 3 and 22  Please note: Lines 1-12 were run initially for all of the questions; Lines 13-23 were run to look for randomized controlled trials related to the treatment of methamphetamine use in adults. |
| **PsycINFO®**  Ovid version rel10.3.0  1985 to May Week 4 2006  Searched May 29, 2006 | 1. exp methamphetamine/  2. (methamphetamine$ or metamphetamine$ or methylamphetamine$).mp.  3. or/1-2  4. limit 3 to (100 childhood or 160 preschool age or 180 school age or 200 adolescence )  5. (adolescen$ or juvenil$ or teen or teens or teenage$ or youth or youths or highschool$ or "high school$").mp.  6. 3 and 5  7. 4 or 6  8. exp Drug Laws/  9. exp "Law (Government)"/  10. exp Legal Processes/  11. exp Policy Making/  12. (arrest$ or legislation or policy or policies or supply or trafficking or law$).ti,ab.  13. or/8-12  14. 3 and 13  15. 7 or 14  16. limit 15 to english language  17. "randomi?ed controlled trial$".mp.  18. "controlled clinical trial$".mp.  19. "random allocation".mp.  20. ("double blind" adj3 method$).mp.  21. ("single blind" adj3 method$).mp.  22. ((clin$ or control$) adj25 trial$).mp.  23. ((singl$ or doubl$ or trebl$ or tripl$) adj25 (blind$ or mask$)).mp.  24. placebo$.mp.  25. random$.mp.  26. or/17-25  27. 3 and 26  Please note: Lines 1-16 were run initially for all of the questions; Lines 17-27 were run to look for randomized controlled trials related to the treatment of methamphetamine use in adults. |
| **International Pharmaceutical Abstracts**  Ovid version rel10.3.0  1970 to April 2006  Searched May 29, 2006 | 1. (methamphetamine$ or metamphetamine$ or methylamphetamine$).mp.  2. 537-46-2.rn.  3. 1 or 2  4. (child$ or schoolchild$ or paediatric$ or pediatric$ or infan$ or adolescen$ or juvenil$ or teen or teens or teenage$ or youth or youths or highschool$ or "high school$").mp.  5. 3 and 4  6. (regulation$ or arrest$ or legislation or policy or policies or legislative or production or supply or trafficking or ((lab$ or laborator$) adj2 (closure$ or seizure$)) or possession or law$ or enforcement).ti,ab.  7. 3 and 6  8. 5 or 7  9. limit 8 to english language  10. limit 9 to human  11. "randomi?ed controlled trial$".mp.  12. "controlled clinical trial$".mp.  13. "random allocation".mp.  14. ("double blind" adj3 method$).mp.  15. ("single blind" adj3 method$).mp.  16. ((clin$ or control$) adj25 trial$).mp.  17. ((singl$ or doubl$ or trebl$ or tripl$) adj25 (blind$ or mask$)).mp.  18. placebo$.mp.  19. random$.mp.  20. or/11-19  21. 3 and 20  22. limit 21 to human  Please note: Lines 1-10 were run initially for all of the questions; Lines 11-22 were run to look for randomized controlled trials related to the treatment of methamphetamine use in adults. |
| **Pascal**  Ovid version rel10.3.0  1987 to April 2006  Searched May 29, 2006 | 1. (methamphetamine$ or metamphetamine$ or methylamphetamine$).mp.  2. Metamfetamine.sh.  3. or/1-2  4. child.sh.  5. adolescent.sh.  6. (child$ or schoolchild$ or paediatric$ or pediatric$ or infan$ or adolescen$ or juvenil$ or teen or teens or teenage$ or youth or youths or highschool$ or "high school$").mp.  7. or/4-6  8. 3 and 7  9. (regulation$ or arrest$ or legislation or policy or policies or legislative or production or supply or trafficking or ((lab$ or laborator$) adj2 (closure$ or seizure$)) or possession or law$ or enforcement).ti,ab.  10. 3 and 9  11. 8 or 10  12. "randomi?ed controlled trial$".mp.  13. "controlled clinical trial$".mp.  14. "random allocation".mp.  15. ("double blind" adj3 method$).mp.  16. ("single blind" adj3 method$).mp.  17. ((clin$ or control$) adj25 trial$).mp.  18. ((singl$ or doubl$ or trebl$ or tripl$) adj25 (blind$ or mask$)).mp.  19. placebo$.mp.  20. random$.mp.  21. or/12-20  22. 3 and 21  Please note: Lines 1-11 were run initially for all of the questions; Lines 12-22 were run to look for randomized controlled trials related to the treatment of methamphetamine use in adults. |
| **EBM Reviews Full Text – CDSR (Cochrane Database of Systematic Reviews), ACP Journal Club, DARE (Database of Abstracts of Reviews of Effects)**  Ovid version rel10.3.0  1st Quarter 2006  Searched May 30, 2006 | 1. methamphetamine$.mp.  2. "randomi?ed controlled trial$".mp.  3. "controlled clinical trial$".mp.  4. "random allocation".mp.  5. ("double blind" adj3 method$).mp.  6. ("single blind" adj3 method$).mp.  7. ((clin$ or control$) adj25 trial$).mp.  8. ((singl$ or doubl$ or trebl$ or tripl$) adj25 (blind$ or mask$)).mp.  9. placebo$.mp.  10. random$.mp.  11. "research design$".mp.  12. "clinical research".mp.  13. (cohort or control$ or observational or prospectiv$ or "time series" or "time-series" or "case comparison" or "case-comparison" or "case referent" or "case-referent" or "cross sectional" or "cross-sectional" or risk$ or effectiveness or "multi center" or "multi centre" or multicenter or multicentre or multisite or "multi site" or "before and after" or predict$ or prevent$).ti,ab.  14. or/2-13  15. 1 and 14 |
| **Global Health**  Ovid version rel10.3.0  1973 to April 2006  Searched May 30, 2006 | 1. methamphetamine$.mp.  2. methylamphetamine$.mp.  3. or/1-2  4. "randomi?ed controlled trial$".mp.  5. "controlled clinical trial$".mp.  6. "random allocation".mp.  7. ("double blind" adj3 method$).mp.  8. ("single blind" adj3 method$).mp.  9. ((clin$ or control$) adj25 trial$).mp.  10. ((singl$ or doubl$ or trebl$ or tripl$) adj25 (blind$ or mask$)).mp.  11. placebo$.mp.  12. random$.mp.  13. "research design$".mp.  14. "clinical research".mp.  15. (cohort or control$ or observational or prospectiv$ or "time series" or "time-series" or "case comparison" or "case-comparison" or "case referent" or "case-referent" or "cross sectional" or "cross-sectional" or risk$ or effectiveness or "multi center" or "multi centre" or multicenter or multicentre or multisite or "multi site" or "before and after" or predict$ or prevent$).ti,ab.  16. or/4-15  17. 3 and 16  18. (child$ or schoolchild$ or paediatric$ or pediatric$ or infan$ or adolescen$ or juvenil$ or teen or teens or teenage$ or youth or youths or highschool$ or "high school$").mp.  19. 17 and 18  20. (regulation$ or arrest$ or legislation or policy or policies or legislative or production or supply or trafficking or ((lab$ or laborator$) adj2 (closure$ or seizure$)) or possession or law$ or enforcement).ti,ab.  21. 17 and 20  22. 19 or 21  23. animal models.sh.  24. 22 not 23  25. or/4-12  26. 3 and 25  Please note: Lines 1-24 were run initially for all of the questions; Lines 25-26 were run to look for randomized controlled trials related to the treatment of methamphetamine use in adults. |
| **PubMed®**  Searched May 30, 2006 for articles from the last 60 days | methamphetamine OR methamphetamine* OR "Amphetamine-Related Disorders"[MeSH] – limit to the last 60 days |
| **Science Citation Index Expanded and Social Sciences Citation Index (via Web of Science®)**  Searched May 30, 2006 | | #16 | #15 OR #13 | | --- | --- | | #15 | #14 AND #11 | | #14 | TS=(regulation* OR arrest* OR legislation OR policy OR policies OR legislative OR production OR supply OR trafficking OR possession OR law* or enforcement) OR TS=(lab*SAME closure*) OR TS=(lab* SAME seizure*) | | #13 | #12 AND #11 | | #12 | TS=(child* OR schoolchild* OR paediatric* OR pediatric* OR infan* OR adolescen* OR juvenil* OR teen OR teens OR teenage* OR youth OR youths OR highschool* OR boy* OR girl*) OR TS=high school* | | #11 | #9 AND #10 | | #10 | TS=(methamphetamine* OR metamfetamine* OR metamphetamine* OR d-methamphetamine* OR dextro-methamphetamine* OR methylamphetamine*) | | #9 | #8 OR #7 OR #6 | | #8 | TS=time series OR TS=time-series OR TS=case comparison OR TS=case-comparison OR TS=case referent OR TS=case-referent OR TS=cross sectional OR TS=cross-sectional OR TS=multi center OR TS=multi centre OR TS=multicenter OR TS=multicentre OR TS=multisite OR TS=multi site OR TS=(before SAME stud*) OR TS=case control stud* OR TS=treatment outcome* | | #7 | TS=(cohort OR observational OR control* OR risk* OR effectiveness OR retrospective OR longitudinal OR predict* OR prevent*) | | #6 | #1 OR #2 OR #3 OR #4 OR #5 | | #5 | TS=randomized controlled trial* OR TS=controlled clinical trial* OR TS=research design OR TS=comparative stud* OR TS=evaluation stud* OR TS=controlled trial* OR TS=follow-up stud* OR TS=prospective stud* | | #4 | TS=random* | | #3 | TS=placebo* | | #2 | TS=clinical trial* | | #1 | TS=(single blind*) OR TS=(double blind*) |   Please note: the preceding search was run initially for all of the questions; the following search was run to look for randomized controlled trials related to the treatment of methamphetamine use in adults.   | #8 | #7 AND #1 | | --- | --- | | #7 | #6 OR #5 OR #4 OR #3 OR #2 | | #6 | TS=randomized controlled trial* OR TS=controlled clinical trial* OR TS=research design OR TS=controlled trial* | | #5 | TS=random* | | #4 | TS=placebo* | | #3 | TS=clinical trial* | | #2 | TS=(single blind*) OR TS=(double blind*) | | #1 | TS=(methamphetamine* OR metamfetamine* OR metamphetamine* OR d-methamphetamine* OR dextro-methamphetamine* OR methylamphetamine*) | |
| **ERIC**  Ovid version rel10.3.0  1966 to April 2006  Searched May 31, 2006 | 1. methamphetamine$.mp.  2. limit 1 to english language |
| **Campbell Library (C2-SPECTR - The Campbell Collaboration Social, Psychological, Educational and Criminological Trials Register and C2-PROT)**  Searched June 7, 2006 | Amphetamines- [Keywords] or Drug-Rehabilitation [Keywords] |
| **Sociological Abstracts**  Searched May 31, 2006 | methylamphetamine* or methamphetamine* |
| **Social Sciences Abstracts**  Searched June 01, 2006 | (ZU "METHAMPHETAMINE") or methamphetamine* |
| **Social Policy & Practice**  Up to March 2006  Searched June 1, 2006 | methamphetamine* |
| **Academic Search Premier**  Searched June 1, 2006 | *Search strategy #1:*  (ZE "METHAMPHETAMINE") or (ZE "METHAMPHETAMINE ABUSE") or methamphetamine* or metamphetamine* or metamfetamine* or d-methamphetamine* or methylamphetamine* or “crystal meth”  and  random* or cohort or control* or observational or prospective* or "time series" or "time-series" or "case comparison" or "case-comparison" or "case referent" or "case-referent" or "cross sectional" or "cross-sectional" or risk* or effectiveness or "multi center" or "multi centre" or multicenter or multicentre or multisite or "multi site" or "before and after" or predict* or prevent*  and  child* or schoolchild* or paediatric* or pediatric* or infan* or adolescent* or juvenil* or teen or teens or teenage* or youth or youths or highschool* or "high school$" or boy* or girl*  *Search strategy #2:*  (ZE "METHAMPHETAMINE") or (ZE "METHAMPHETAMINE ABUSE") or methamphetamine* or metamphetamine* or metamfetamine* or d-methamphetamine* or methylamphetamine* or “crystal meth”  and  random* or cohort or control* or observational or prospective* or "time series" or "time-series" or "case comparison" or "case-comparison" or "case referent" or "case-referent" or "cross sectional" or "cross-sectional" or risk* or effectiveness or "multi center" or "multi centre" or multicenter or multicentre or multisite or "multi site" or "before and after" or predict* or prevent*  and  regulation* or arrest* or legislation or policy or policies or legislative or production or supply or trafficking or (lab* N3 closure*) or (lab* N3 seizure*) or possession or law* or enforcement |
| **Sage Fulltext Collections (CSA)**  Searched June 1, 2006 | *Search strategy #1:*  methamphetamine* or metamphetamine* or methylamphetamine* or crystal meth  and  random* or cohort or control* or observational or prospective* or "time series" or "time-series" or "case comparison" or "case-comparison" or "case referent" or "case-referent" or "cross sectional" or "cross-sectional" or risk* or effectiveness or "multi center" or "multi centre" or multicenter or multicentre or multisite or "multi site" or "before and after" or predict* or prevent*  and  child* or schoolchild* or paediatric* or pediatric* or infan* or adolescent* or juvenil* or teen or teens or teenage* or youth or youths or highschool* or "high school$" or boy* or girl*  *Search strategy #2:*  methamphetamine* or metamphetamine* or methylamphetamine* or crystal meth  and  random* or cohort or control* or observational or prospective* or "time series" or "time-series" or "case comparison" or "case-comparison" or "case referent" or "case-referent" or "cross sectional" or "cross-sectional" or risk* or effectiveness or "multi center" or "multi centre" or multicenter or multicentre or multisite or "multi site" or "before and after" or predict* or prevent*  and  regulation* or arrest* or legislation or policy or policies or legislative or production or supply or trafficking or (lab* within 3 closure*) or (lab* within 3 seizure*) or possession or law* or enforcement |
| **Psychology and Behavioural Sciences Collection**  Searched June 1, 2006 | (ZU "METHAMPHETAMINE") or (ZU "METHAMPHETAMINE -- PSYCHOLOGICAL ASPECTS") or (ZU "METHAMPHETAMINE ABUSE") or (ZU "METHAMPHETAMINE ABUSE -- SOCIAL ASPECTS") or methamphetamine* or metamphetamine* or d-methamphetamine* or methylamphetamine*  not  ((ZU "RATS") or (ZU "RATS AS LABORATORY ANIMALS") or (ZU "RATS AS LABORATORY ANIMALS -- BEHAVIOR") or (ZU "RATS AS LABORATORY ANIMALS -- PHYSIOLOGY")) or ((ZU "MICE") or (ZU "MICE AS LABORATORY ANIMALS") or (ZU "MICE AS LABORATORY ANIMALS -- BEHAVIOR")) or TI ("RATS") or TI (“MICE”) |
| **PAIS (Public Affairs Information Service) International**  Searched June 2, 2006 | methamphetamine* |
| **International Digest Of Health Legislation (WHO)**  Searched June 2, 2006 | methamphetamine* |
| **Criminal Justice Abstracts**  Searched June 5, 2006 | methamphetamine* or methylamphetamine* or metamphetamine* |
| **LegalTrac**  Searched June 5, 2006 | "methamphetamine_laws, regulations and rules" or "methamphetamine_cases" in Basic Search |
| **Index to Legal Periodicals & Books Fulltext**  Searched June 7, 2006 | methamphetamine* |
| **Index to Canadian Legal Literature**  Searched June 7, 2006 | Drug Abuse, Drug Traffic, Narcotics Control, Substance Abuse (under subject) |
| **AGIS Plus Text**  Searched June 6, 2006 | methamphetamine* |
| **Current Legal Information**  Searched June 6, 2006 | methamphetamine* or methylamphetamine* or amphetamine* |
| **EUR-LEX**  Searched June 6, 2006 | methamphetamine or methamphetamines |
| **CBCA Business**  Searched June 5, 2006 | methamphetamine* or metamphetamine* |
| **ABI Inform®**  Searched June 2, 2006 | methamphetamine* or metamphetamine*  and  random* or cohort or control* or observational or prospective* or "time series" or "time-series" or "case comparison" or "case-comparison" or "case referent" or "case-referent" or "cross sectional" or "cross-sectional" or risk* or effectiveness or "multi center" or "multi centre" or multicenter or multicentre or multisite or "multi site" or "before and after" or predict* or prevent* or regulation* or arrest* or legislation or policy or policies or legislative or production or supply or trafficking or "lab* closure*" or "lab* seizure*" or possession or law* or enforcement |
| **First Nations Pediodical Index**  Searched June 6, 2006 | crystal meth |
| **Native Health Research Database**  Searched June 6, 2006 | methamphetamine or methamphetamines or crystal meth |
| **Bibliography of Native North Americans**  Searched June 21, 2006 | methamphetamine* |

**Grey Literature Databases**

| **Grey Literature Databases Searched** (and website addresses, if applicable) | **Search Strategies** |
| --- | --- |
| **BioMedCentral**  (http://www.biomedcentral.com)  Searched 29.6.06 | Methamphetamine* AND (youth OR adolescent* OR “young adult” OR child*) |
| **Canada Institute for Scientific and Technical Information (CISTI)** (http://cat.cisti-icist.nrc-cnrc.gc.ca/search)  Searched 16.6.06 | ((met*amphetamine* OR met*amfetamine*) AND stud**)  ((met*amphetamine* OR met*amfetamine*) AND child**)  ((met*amphetamine* OR met*amfetamine*) AND adolescent*)  ((met*amphetamine* OR met*amfetamine*) AND youth")  ((met*amphetamine* OR met*amfetamine*) AND "young people") |
| **Canadian Research Index**  Searched 23.5.06; 30.6.06 | (methamphetamine OR metamfetamine OR methylamphetamine )  crystal meth  addiction AND methamphetamine  precursor* AND (policy OR legislation OR control)  (drug OR methamphetamine) AND precursor* |
| **CRISP (Computer Retrieval of Information on Scientific Projects)**  (http://crisp.cit.nih.gov/)  Searched 26.5.06 | (methamphetamine OR methylamphetamine OR metamfetamine OR metamphetamine OR methamphetamines OR methylamphetamines OR metamfetamines OR metamphetamines )  methamphetamine AND youth  methamphetamine AND adolescents  methamphetamine AND children |
| **Curtin University of Technology, National Drug Research Institute: Indigenous Australian Alcohol & Other Drug Databases**  (http://www.db.ndri.curtin.edu.au)  Searched 23.6.06 | A pre-programmed search with drop down menus to guide the user to find answers to drug use, drug harms, associated factors and determinants of drug misuse, interventions, and recommendations, all with a focus on Australian Indigenous people. |
| **E-Brary**  Searched 1.6.06 | methamphetamine AND (children OR youth OR "young adults" OR families)  “crystal meth” AND (children OR youth OR "young adults" OR families) |
| **Health Research Projects in Progress**  (http://wwwcf.nlm.nih.gov/hsr_project/home_proj.cfm)  Searched 16.6.06 | ((methamphetamine OR methamphetamines OR methylamphetamine OR methylamphetamines OR metamphetamine OR metamphetamines OR metamfetamine OR metamfetamines) AND (study OR studies)) |
| **Index to Theses of Great Britain and Ireland**  (http://www.theses.com)  Searched 22.6.06 | meth*amphetamine* AND (child* OR adolescents)  meth*amphetamine* OR metam*etamine* |
| **International Digest of Health Legislation**  (http://www.who.int/idhl)  Searched 14.6.06 | Keyword Search:  methamphetamine  alternative methamphetamine spellings not recognized  controlled substance  drug abuse  precursors  By Subject (Select from list):  XC. Drug abuse (narcotics, psychotropic drugs, and other dependence-producing drugs)  Then select country of interest from list. |
| **National Institute on Alcohol Abuse and Alcoholism (NIAAA)**  **ETOH: Alcohol and Alcohol Problems Science Database**  (http://etoh.niaaa.nih.gov)  Searched 19.5.06 | methamphetamine AND children  methamphetamine AND adolescents  methamphetamine AND youth  methamphetamine AND study  methamphetamine AND studies  methamphetamine AND policy  methamphetamine AND legislation  precursor control |
| **National Library of Medicine's LocatorPlus**  (http://locatorplus.gov)  Searched 27.6.06 | (methamphetamine OR methamphetamines) AND (child OR children OR adolescent OR adolescents OR youth)  (methamphetamine OR methamphetamines) AND (study OR studies) |
| **Networked Digital Library of Theses and Dissertations**  (http://hercules.vtls.com/cgi-bin/ndltd/chameleon)  Searched 5.6.06 | Subject search: methamphetamine OR methamphetamines |
| **NLM Gateway**  (http://gateway.nlm.nih.gov)  Searched 27.6.06 | methamphetamine* AND (adolescent* OR "young adult*" OR child* OR youth) AND prevention  methamphetamine* and "harm reduction"  methamphetamine* and "best practice*"  methamphetamine* and treatment |
| **OCLC ProceedingsFirst**  Searched 20.5.06 | (methamphetamine OR metamfetamine OR methylamphetamine ) AND stud* |
| **OCLC PapersFirst**  Searched 20.5.06 | (methamphetamine OR metamfetamine OR methylamphetamine ) AND stud* |
| **Official Document System of the UN (ODS)**  (http://documents.un.org)  Searched 30.6.06 | ((methamphetamine OR methamphetamines) AND (child OR children OR youth OR “young adult” OR “young adults”) AND “best practices”)  ((methamphetamine OR methamphetamines) AND (child OR children OR youth OR “young adult” OR “young adults”) AND (treatment OR treatments OR rehabilitation OR recovery))  ((methamphetamine OR methamphetamines) AND (child OR children OR youth OR “young adult” OR “young adults”) AND (prevention OR prevent)) |
| **Proquest Dissertations and Abstracts**  Searched 2.6.06 | (child* AND ((drug OR substance) AND (misuse ORabuse)))  (((child* or adolescent* or youth) and ((drug or substance) and abuse)))  (polic* AND ((drug OR substance) AND (misuse ORabuse))) |
| **SciELO (Scientific Electronic Library Online)**  (http://www.scielo.org)  Searched 19.5.06 | Thesaurus: preferred term is amphetamine or amphetamine like substances for methylamphetamine; no other spelling of methamphetamine recognized  all word search:  amphetamine amphetamines  amphetamine like substances |
| **Theses Canada Portal**  (http://www.collectionscanada.ca/thesescanada)  Searched 22.6.06 | (methamphetamine OR (methamphetamines) NOT animal |
| **VHL** **(Virtual Health Library of El Salvador, Costa Rica, Argentina, Columbia, Bolivia, Brazil, Cuba, Honduras, Trinidad/Tobago, Uruguay, Venezuela, Spain, Peru)**  (http://www.bireme.br/bvs/E/ehome.htm)  Searched 2.6.06  A Library Portal searching:  LILACS - Latin American and Caribbean Health Sciences  MEDLINE 1996-2006 - International Database for Medical  Literature  MEDLINE 1966-1995 - International Database for Medical  Literature  ADOLEC - Literature on Adolescence Health  BBO - Brazilian Bibliography of Dentistry  BDENF - Nursing Database  BIOETHICS - Data base of the Regional Program on Bioethics  PAHO/WHO  DESASTRES - Disaster Documentation Center Collection  HOMEOINDEX - Homeopathy Brazilian Bibliography  LEYES - Latin American and Caribbean Basic Health Legislation  MEDCARIB - Caribbean Health Sciences Literature  REPIDISCA - Sanitary Engineering and Environmental Sciences | (methamphetamine$ OR metamfetamine$) AND (adolescent$ OR child$)  Crystal Meth |
| **WHOLIS (Library and Information Network of the World Health Organization [WHO])**  (http://dosei.who.int)  Searched 2.6.06 | methamphetamine$ AND stud$  **amphetamine$** |

**Websites**

| **Websites Searched - Government Websites** | | |
| --- | --- | --- |
| **Site** | **Search Date** | **Website Address** |
| Administrative Office of the Courts, Center for Families, Children & the Courts | 19.6.06 | <http://www.courtinfo.ca.gov/programs/cfcc> |
| Australian Government Publications | 12.6.06 | [http://www.publications.gov.au](http://www.publications.gov.au/) |
| Australian Government. Institute of Family Studies | 12.6.06 | <http://www.aifs.gov.au/na2.htm> |
| Canada Gazette | 29.6.06 | [http://canadagazette.gc.ca](http://canadagazette.gc.ca/) |
| Canadian Federal Publication Locator | 24.5.06 | <http://www.collectionscanada.ca/7/5/index-e.html> |
| City of London, UK | 6.7.06 | [http://www.london.gov.uk](http://www.london.gov.uk/) |
| City of Vancouver  Four Pillars Drug Strategy | 26.6.06 | <http://www.city.vancouver.bc.ca/fourpillars/index.htm> |
| Criminal Intelligence Service of Canada | 25.5.06 | [http://www.cisc.gc.ca](http://www.cisc.gc.ca/) |
| Department of Justice Canada | 29.6.06 | <http://laws.justice.gc.ca/en/C-38.8/SOR-2002-359/index.html> |
| Directory of Health Organizations Online | 19.5.06 | [http://dirline.nlm.nih.gov](http://dirline.nlm.nih.gov/) |
| FirstGov: US Government's Official Web Portal | 30.6.06 | [http://www.firstgov.gov](http://www.firstgov.gov/) |
| Government of Alberta | 24.5.06 | [http://www.gov.ab.ca](http://www.gov.ab.ca/) |
| Government of British Columbia | 14.6.06 | [http://www.gov.bc.ca](http://www.gov.bc.ca/) |
| Government of Canada | 24.5.06 | <http://canada.gc.ca/main_e.html> |
| Government of Canada DSP | 24.5.06 | <http://dsp-psd.pwgsc.gc.ca/index-e.html> |
| Government of Canada Publications | 24.5.06 | <http://publications.gc.ca/control/simplePublicSearch> |
| Government of Manitoba | 12.6.06 | [http://www.gov.mb.ca](http://www.gov.mb.ca/) |
| Government of Nova Scotia | 16.6.06 | [http://www.gov.ns.ca](http://www.gov.ns.ca/) |
| Government of Nova Scotia. Policy Watch | 16.6.06 | <http://www.gov.ns.ca/heal/policywatch> |
| Government of Ontario | 24.5.06 | [http://gov.on.ca](http://gov.on.ca/) |
| Government of the Yukon | 13.6.06 | [http://gov.yk.ca](http://gov.yk.ca/) |
| GrayLit Network | 19.5.06 | [http://graylit.osti.gov](http://graylit.osti.gov/) |
| Home Office. Drug Prevention Advisory Service | 6.7.06 | <http://www.homeoffice.gov.uk/drugs> |
| Indian and Northern Affairs Canada | 25.5.06 | [http://srch.ainc-inac.gc.ca](http://srch.ainc-inac.gc.ca/) |
| LEGISinfo: a research tool for finding information on legislation currently before Canada’s Parliament | 30.6.06 | <http://www.parl.gc.ca/LEGISINFO/index.asp?Language=E&Session=14&List=search> |
| Library of Congress THOMAS: a research tool for finding current and historic information on US federal legislation | 29.6.06 | [http://thomas.loc.gov](http://thomas.loc.gov/) |
| National Criminal Justice Reference Service (NCJRS) | 13.6.06 | [http://www.ncjrs.gov](http://www.ncjrs.gov/) |
| National Drug Policy New Zealand | 6.6.06 | [http://www.ndp.govt.nz](http://www.ndp.govt.nz/) |
| National Guideline Clearinghouse | 16.6.06 | [http://www.guideline.gov](http://www.guideline.gov/) |
| Office of National Drug Control Policy (ONDCP) | 13.6.06 | [http://www.whitehousedrugpolicy.gov](http://www.whitehousedrugpolicy.gov/) |
| Organization of the American States (OAS) | 26.5.06 | <http://www.oas.org/main/english> |
| Parliament of Canada | 24.5.06 | [http://www.parl.gc.ca](http://www.parl.gc.ca/common/index.asp?Language=E) |
| Public Safety and Emergency Preparedness Canada | 24.5.06 | [http://www.psepc.gc.ca](http://www.psepc.gc.ca/) |
| RCMP | 29.5.06 | [http://rcmp-grc.gc.ca](http://rcmp-grc.gc.ca/) |
| Statistics Canada | 25.5.06 | <http://www.statcan.ca/cgi-bin/statcomment.pl> |
| Substance Abuse and Mental Health Data Archive | 5.7.06 | <http://www.icpsr.umich.edu/SAMHDA> |
| US Department of Justice | 25.5.06 | [http://www.usdoj.gov](http://www.usdoj.gov/) |
| US Department of Health & Human Services | 14.6.06 | [http://www.hhs.gov](http://www.hhs.gov/) |
| US Department of Health & Human Services. Administration for Children and Families. National Clearinghouse on Child Abuse and Neglect Information | 14.6.06 | [http://nccanch.acf.hhs.gov](http://nccanch.acf.hhs.gov/) |
| US Department of Justice. Office for Victims of Crime | 25.5.06 | <http://www.ojp.usdoj.gov/ovc> |
| US Department of State | 30.6.06 | [http://www.state.gov](http://www.state.gov/) |
| US Drug Enforcement Administration (DEA) | 25.5.06 | [http://www.dea.gov](http://www.dea.gov/) |
| **Websites Searched - Health Websites (Government, Research Centres, etc.)** | | |
| **Site** | **Search**  **Date** | **Website Address** |
| Addiction Research Foundation (Canada) | 26.5.06 | [http://www.arf.org](http://www.arf.org/) |
| Addiction Technology Transfer Center Network | 27.6.06 | <http://www.ceattc.org/index.asp> |
| Addictions Foundation of Manitoba | 26.6.06 | [http://www.afm.mb.ca](http://www.afm.mb.ca/) |
| Alberta College of Pharmacists | 19.5.06 | [https://pharmacists.ab.ca](https://pharmacists.ab.ca/) |
| American Psychiatric Association | 27.6.06 | [http://www.psych.or](http://www.psych.or/) |
| Asian Harm Network | 27.6.06 | [http://www.ahrn.net](http://www.ahrn.net/) |
| Australian Drug Information Network | 12.6.06 | [http://www.adin.com.au](http://www.adin.com.au/) |
| Australian Government Department of Health and Aging | 12.6.06 | [http://www.health.gov.au](http://www.health.gov.au/) |
| Australian Institute of Health and Welfare | 12.6.06 | [http://www.aihw.gov.au](http://www.aihw.gov.au/) |
| British Columbia Medical Association | 6.6.06 | [www.bcma.org](http://www.bcma.org/) |
| Canadian Centre for Substance Abuse | 1.6.06 | [http://www.ccsa.ca](http://www.ccsa.ca/) |
| Canadian Harm Reduction Network | 8.6.06 | [http://www.canadianharmreduction.com](http://www.canadianharmreduction.com/) |
| Canadian Health Services Research Foundation | 13.6.06 | <http://www.chsrf.ca/home_e.php> |
| Canadian Institute for Health Information (CIHI) | 12.6.06 | <http://secure.cihi.ca/cihiweb/dispPage.jsp?cw_page=home_e> |
| Canadian Institutes of Health Research | 26.6.06 | [http://www.cihr-irsc.gc.ca](http://www.cihr-irsc.gc.ca/) |
| Canadian Institutes of Health Research heath-evidence.ca | 26.6.06 | [http://health-evidence.ca](http://health-evidence.ca/) |
| Canadian Medical Association | 19.5.06 | [http://www.cma.ca](http://www.cma.ca/) |
| Centre for Addiction and Mental Health | 2.6.06 | [http://www.camh.net](http://www.camh.net/) |
| Centre for Addictions Research of BC | 8.6.06 | [www.silink.ca](http://www.silink.ca/) |
| Centre for Social and Health Outcomes Research | 6.6.06 | [http://www.shore.ac.nz](http://www.shore.ac.nz/) |
| **Clinical Medicine and Health Research NetPrints** | 2.6.06 | [http://clinmed.netprints.org](http://clinmed.netprints.org/) |
| Department of Health and Children (Ireland) | 26.6.06 | [http://www.dohc.ie](http://www.dohc.ie/) |
| DrugScope : Informing Policy Reducing Risk | 23.6.06 | <http://drugscope.soutron.com/home.asp> |
| Government of Alberta. Alberta Alcohol and Drug Abuse Commission | 24.5.06 | [http://www.aadac.com](http://www.aadac.com/) |
| Government of British Columbia. Ministry of Health. Mental Health and Addictions | 19.5.06 | <http://www.health.gov.bc.ca/mhd> |
| Gulf Coast Addiction Technology Transfer Center | 27.6.06 | <http://www.utexas.edu/research/cswr/gcattc> |
| Harm Reduction Journal | 8.6.06 | [http://www.harmreductionjournal.com](http://www.harmreductionjournal.com/) |
| Health Canada | 29.5.06 | [http://www.hc-sc.gc.ca](http://www.hc-sc.gc.ca/) |
| Health Services/Technology Assessment Text | 16.6.06 | <http://www.ncbi.nlm.nih.gov/books/bv.fcgi?rid=hstat> |
| Here To Help | 8.6.06 | [http://www.heretohelp.bc.ca](http://www.heretohelp.bc.ca/) |
| HIT- delivers effective interventions on drugs, community safety and other public health concerns | 23.6.06 | <http://www.hit.org.uk/default.asp> |
| Information Centre on Aboriginal Health | 13.6.06 | [www.icah.ca](http://www.icah.ca/) |
| International Harm Reduction | 8.6.06 | [http://www.ihra.net](http://www.ihra.net/) |
| Journal of Medical Internet Research | 23.6.06 | [http://www.jmir.org](http://www.jmir.org/) |
| McCreary Centre Society | 8.6.06 | [www.mcs.bc.ca](http://www.mcs.bc.ca/) |
| MedLinePlus | 13.6.06 | [http://medlineplus.gov](http://medlineplus.gov/) |
| National Center on Substance Abuse and Child Welfare (NCSACW) | 5.6.06 | <http://www.ncsacw.samhsa.gov/products.asp> |
| National Clearinghouse for Alcohol and Drug Information (NCADI) | 5.6.06 | [http://ncadi.samhsa.gov](http://ncadi.samhsa.gov/) |
| National Framework for Action to Reduce the Harms Associated with Alcohol and Other Drugs and Substances in Canada | 29.6.06 | <http://www.nationalframework-cadrenational.ca/index_e.php?orderid_top=2> |
| National Health Services UK | 26.6.06 | [http://www.nhs.uk](http://www.nhs.uk/) |
| National Institute on Drug Abuse (NIDA) | 29.6.06 | <http://www.drugabuse.gov/index.html> |
| NHS National Institute for Health and Clinical Excellence (NICE) | 26.6.06 | <http://www.nice.org.uk/page.aspx?o=home> |
| NHS National Treatment Agency for Substance Misuse (NTA) | 26.6.06 | [http://www.nta.nhs.uk](http://www.nta.nhs.uk/) |
| NOAH: New York Online Access to Health | 19.5.06 | [http://www.noah-health.org](http://www.noah-health.org/) |
| Northern Family Health Society | 15.6.06 | [http://www.nfhs-pg.org](http://www.nfhs-pg.org/) |
| Pan American Health Organization | 2.6.06 | [http://www.paho.org](http://www.paho.org/) |
| Project Cork | 1.6.06 | [http://www.projectcork.org](http://www.projectcork.org/) |
| Provincial Centre of Excellence for Child and Youth Mental Health at CHEO | 26.6.06 | <http://www.alafinepointe.ca/kec/documents/CPGannotbib.pdf> |
| Robert Wood Johnson Foundation | 8.6.06 | [http://www.rwjf.org](http://www.rwjf.org/) |
| Treatment Improvement Exchange | 16.6.06 | [http://www.treatment.org](http://www.treatment.org/) |
| Trimbos-instituut: Netherlands Institute of Mental Health and Addiction | 27.6.06 | <http://www.trimbos.nl/default37.html> |
| Turning Point Alcohol & Drug Centre | 28.6.06 | [http://www.turningpoint.org.au](http://www.turningpoint.org.au/) |
| University of New South Wales. National Drug & Alcohol Research Centre (NDARC) | 23.6.06 | <http://ndarc.med.unsw.edu.au/ndarcweb.nsf/page/home> |
| US Department of Health and Human Services. Substance Abuse & Mental Health Services Administration (SAMHSA) | 5.6.06 | [http://www.samhsa.gov](http://www.samhsa.gov/) |
| US Department of Human and Health Services. Indian Health Service (IHS) | 9.5.06 | [http://www.ihs.gov](http://www.ihs.gov/) |
| US Department of Human Health & Services. Office of Applied Studies (OAS) | 1.6.06 | <http://oas.samhsa.gov/nsduh.htm> |
| Utah Addictions Center | 16.6.06 | <http://uuhsc.utah.edu/uac> |
| Washington State Department of Health | 1.6.06 | [http://www.doh.wa.gov/ehp/ts/CDL/resources.htm#DEC](http://www.doh.wa.gov/ehp/ts/CDL/resources.htm#DEC) |
| World Health Organization (WHO) | 2.6.06 | [http://www.who.int](http://www.who.int/) |
| **Websites Searched – MA-related Organizations and Agencies** | | |
| **Site** | **Search**  **Date** | **Website Address** |
| Crystal Meth in Edmonton | 13.6.06 | <http://www.edmonton.ca/CityGov/CommServices/SaferCitiesReportOnCrystalMethFebruary04.pdf> |
| CrystalRecovery.com | 8.6.06 | [www.crystalrecovery.com](http://www.crystalrecovery.com/) |
| Government of British Columbia. Ministry of Public Safety and Solicitor General. Crystal Meth Secretariat | 12.6.06 | <http://www.pssg.gov.bc.ca/crystalmeth> |
| Manitoba Meth Strategy | 12.6.06 | <http://www.gov.mb.ca/healthyliving/meth.html> |
| Methamphetamine Treatment Project | 5.6.06 | [http://www.methamphetamine.org](http://www.methamphetamine.org/) |
| Methamphetamine, ecstasy and BZP in New Zealand: An annotated bibliography | 5.6.06 | <http://www.chamberlain.net.nz/research/introduction1.htm> |
| Methfacts.org | 8.6.06 | [http://methfacts.org](http://methfacts.org/) |
| Meth-Free Mesa County | 1.6.06 | [http://methfree.mesacounty.us](http://methfree.mesacounty.us/) |
| MethResources.gov | 1.6.06 | [http://www.methresources.gov](http://www.methresources.gov/) |
| National Alliance for Model State Drug Laws (US) | 29.6.06 | <http://www.natlalliance.org/publications.asp> |
| The Anti-Meth Site (formerly the Koch Crime Institute) | 8.6.06 | [http://www.kci.org](http://www.kci.org/) |
| **Websites Searched – Other** | | |
| **Site** | **Search**  **Date** | **Website Address** |
| Aboriginal Healing Foundation | 13.6.06 | [http://www.ahf.ca](http://www.ahf.ca/) |
| Aboriginal Youth Network | 13.6.06 | <http://ayn.ca/AYNHome.aspx> |
| American Library Association Gray Literature Page | 19.6.06 | [http://www.ala.org/ala/acrl/acrlpubs/ crlnews/backissues2004/march04/graylit.htm](http://www.ala.org/ala/acrl/acrlpubs/crlnews/backissues2004/march04/graylit.htm) |
| American Statistical Association | 6.7.06 | [http://www.amstat.org](http://www.amstat.org/) |
| Asia Pacific Foundation | 25.5.06 | [http://www.asiapacificresearch.ca](http://www.asiapacificresearch.ca/) |
| Australian Clearinghouse for Youth Studies | 12.6.06 | [http://www.acys.info](http://www.acys.info/) |
| Australian Legal Information Institute | 12.6.06 | [http://www.austlii.edu.au](http://www.austlii.edu.au/) |
| Australian Social Science Data Archive | 12.6.06 | [http://assda.anu.edu.au](http://assda.anu.edu.au/) |
| Bell Canada Child Welfare Research Unit | 14.6.06 | <http://www.rowwest.com/childwelfare> |
| Canadian Centre for Policy Alternatives | 25.5.06 | [http://www.policyalternatives.ca](http://www.policyalternatives.ca/) |
| Canadian Council on Social Development | 25.5.06 | [http://www.ccsd.ca](http://www.ccsd.ca/) |
| Carlton University’s Country Indicators of Foreign Policy | 19.5.06 | <http://www.carleton.ca/cifp> |
| Catalaw | 25.5.06 | [http://www.catalaw.com](http://www.catalaw.com/) |
| Colorado Alliance for Drug Endangered Children | 21.6.06 | [http://www.colodec.org/decpapers/decpapers.htm#childabuseneglect](http://www.colodec.org/decpapers/decpapers.htm#childabuseneglect) |
| Conseil National du Sida (National AIDS Council, France) | 30.6.06 | <http://www.cns.sante.fr/web_sida/index2.htm> |
| Council of Yukon First Nations | 13.6.06 | <http://www.theyukon.ca/dbs/cyfn/index.cfm> |
| Curtin University of Technology, National Drug Research Institute | 23.6.06 | [http://www.db.ndri.curtin.edu.au](http://www.db.ndri.curtin.edu.au/) |
| DrugInfo Clearinghouse | 12.6.06 | [http://www.druginfo.adf.org.au](http://www.druginfo.adf.org.au/) |
| Drug Policy Alliance | 28.6.06 | [http://www.drugpolicy.org](http://www.drugpolicy.org/) |
| Eur-Lex : The portal to European Union Law | 29.6.06 | <http://europa.eu.int/eur-lex/en/index.html> |
| EUROPA: Gateway to EU | 25.5.06 | <http://europa.eu/index_en.htm> |
| European Legal Database on Drugs | 29.6.06 | [http://eldd.emcdda.europa.eu](http://eldd.emcdda.europa.eu/) |
| European Monitoring Centre for Drugs and Drug Addiction (EMCCDA) | 15.6.06 | [http://www.emcdda.europa.eu](http://www.emcdda.europa.eu/) |
| **Fade: The North West Grey Literature Service** | 19.5.06 | [http://www.fade.nhs.uk](http://www.fade.nhs.uk/) |
| Foundation for Alcohol and Drug Education | 6.6.06 | [http://www.fade.org.nz](http://www.fade.org.nz/) |
| Fraser Institute | 25.5.06 | [http://www.fraserinstitute.ca](http://www.fraserinstitute.ca/) |
| Geopium.org | 29.6.06 | [http://www.pa-chouvy.org](http://www.pa-chouvy.org/) |
| GreySource | 19.5.06 | <http://www.greynet.org/greysourceindex.html> |
| International Society for Prevention of Child Abuse and Neglect | 14.6.06 | [http://www.ispcan.org](http://www.ispcan.org/) |
| Journal of Medical Internet Research | 23.6.06 | [http://www.jmir.org](http://www.jmir.org/) |
| Liu Institute for Global Issues | 25.5.06 | [http://www.ligi.ubc.ca](http://www.ligi.ubc.ca/) |
| McCreary Centre Society | 8.6.06 | [http://www.mcs.bc.ca](http://www.mcs.bc.ca/) |
| Media Awareness Project (MAP Inc.) | 8.6.06 | [http://www.mapinc.org](http://www.mapinc.org/) |
| National District Attorneys Association (NDAA) | 14.6.06 | [http://www.ndaa-apri.org](http://www.ndaa-apri.org/) |
| Nechi Training, Research and Health Promotions Institute | 13.6.06 | <http://www.nechi.com/research/main.php> |
| New York Academy of Medicine Grey Literature Report | 2.6.06 | <http://www.nyam.org/library/grey.shtml> |
| Organized Crime Agency of British Columbia (OCABC) | 14.6.06 | <http://www.ocabc.org/press_releases/021402.html> |
| **Qualitative European Drug Research** | 15.6.06 | [http://qed.emcdda.europa.eu](http://qed.emcdda.europa.eu/) |
| RAND | 15.6.06 | [http://www.rand.org](http://www.rand.org/) |
| Synthetic Drugs in Canada | 13.6.06 | [http://www.natlalliance.org/pdfs/Cynthia%20Sunstrom.pdf](http://www.natlalliance.org/pdfs/Cynthia Sunstrom.pdf) |
| Turtle Island | 8.6.06 | [http://www.turtleisland.org](http://www.turtleisland.org/) |
| University of Glasgow Centre for Drug Misuse Research | 23.6.06 | <http://www.gla.ac.uk/centres/drugmisuse> |
| UN Office of Drugs and Crime | 29.6.06 | <http://www.unodc.org/unodc/index.html> |
| **UNICEF** | 15.6.06 | [http://www.unicef.org](http://www.unicef.org/) |
| Urban Native Youth Association | 6.6.06 | [http://www.unya.bc.ca](http://www.unya.bc.ca/) |
| Utah Addictions Center | 16.6.06 | <http://uuhsc.utah.edu/uac> |
| www.streetdrugs.org | 25.5.06 | <http://www.streetdrugs.org/methamphetamine.htm> |
