# Supplementary material for: Risk factors for methamphetamine use in youth: a systematic review
Source: BMC Pediatr. 2008 Oct 28;8:48. doi: 10.1186/1471-2431-8-48 (PMC2588572; doi:10.1186/1471-2431-8-48)
Supplement: Additional File 2 — Table1. Risk factors for methamphetamine use: description of the population. This file contains a table. [file 1471-2431-8-48-S2.doc]

Table 1. Risk factors for methamphetamine use: description of the population

| **Study**  **Year** | **Country** | **Source of Cases** | **Number of Cases** | **Description of Cases** | **Source of Comparisons** | **Number of Comparisons** | **Description of Comparisons** |
| --- | --- | --- | --- | --- | --- | --- | --- |
| Low-risk youth as the comparison group* | | | | | | | |
| Lampinen 2006 | Canada | Schools | 27 | MA users in grades 6-12 from 6 Vancouver and Victoria schools selected for heterogeneity of income, race, and geographic location | Schools | 580 | Non-MA-users in grades 6-12 from 6 Vancouver and Victoria schools selected for heterogeneity of income, race, and geographic location |
| Oetting 2000 | USA | Schools | 15,730 | MA users from 300 high schools in 30 different states | Schools | 62,985 | Students from 300 high schools in 30 different states not using MA |
| Sattah  2002 | Thailand | Schools | 500 | MA using students that attend 1 of 3 vocational schools in Chiang Rai Province | Schools | 1,225 | Students not using MA that attend 1 of 3 vocational schools in Chiang Rai Province |
| Yen  2006 | Taiwan | Juvenile detention center | 200 | Adolescent detainees using MA from 2 juvenile detention centers | Schools | 400 | Age and sex matched students that do not use MA from 2 high schools and 3 vocational schools |
| Yen  2004 | Taiwan | Juvenile detention center | 85 | Adolescent MA users from a detention center in southern Taiwan | Schools | 170 | Sex matched adolescents from 1 high school and 3 vocational schools who deny MA use |
| **High-risk youth as the comparison group** | | | | | | | |
| Kim  2002 | USA | Juvenile home | 485 | MA using adolescents interviewed after admission to 7 different program sites | Juvenile home | 4,159 | Non-MA using adolescents interviewed after admission to 7 different program sites |
| Miura 2006 | Japan | Juvenile home | 1,362 | Adolescents admitted to the Nagoya Juvenile Classification Home with a history of MA use | Juvenile home | 1,269 | Adolescents admitted to the Nagoya Juvenile Classification Home with no history of MA use |
| Palmer 2005 | USA | Treatment | 20 | MA using adolescents admitted to a residential treatment program | Treatment | 40 | 20 hallucinogen addicts and 20 cannabis addicts consecutively admitted to a residential treatment program (matched for age and sex) |
| Rawson 2005 | USA | Out patient treatment | 90 | Substance abusing adolescents who use MA | Out patient treatment | 215 | Substance abusing adolescents who do not use MA |
| Shillington 2005 | USA | Community drop in center | 48 | At risk 14-24 year olds (Hispanic, gay, lesbian, bisexual, or transgender (GLBT) youth, homeless, runaways) who use MA and have dropped in at the center on ≤2 occasions | Community drop in center | 137 | At risk 14-24 year old youth (Hispanic, GLBT youth, homeless, runaways) who use a combination of cigarettes, alcohol, marijuana, and ecstasy and have dropped in at the center on ≤2 occasions |
| Shillington 2003 | USA | Treatment | 1,313 | Adolescent MA users admitted to contracted county treatment | Treatment | 3,420 | Adolescent drug users admitted to contracted county treatment not using MA |
| Uchida 1995 | Japan | Juvenile home | 47 | Adolescent MA users incarcerated in a juvenile home | Juvenile home | 47 | Adolescents non MA users incarcerated in a juvenile home (matched for day of incarceration) |

*Youth who did not use illicit drugs; **Youth who abused illicit drugs other than MA or were in a juvenile detention center.

MA=methamphetamine MA=methamphetamine.
