# Supplementary material for: Risk factors for methamphetamine use in youth: a systematic review
Source: BMC Pediatr. 2008 Oct 28;8:48. doi: 10.1186/1471-2431-8-48 (PMC2588572; doi:10.1186/1471-2431-8-48)
Supplement: Additional File 3 — Table2. Risk factors for methamphetamine use: description of the risk factors. The file contains a table. [file 1471-2431-8-48-S3.doc]

Table 2. Risk factors for methamphetamine use: description of the risk factors

| **Study,**  **Year** | **MA Use** | **Primary Risk Factor** | **Description of Primary Risk Factors Reported in the Study** | **Additional Risk Factors Reported in the Study** | **Authors’ Conclusions** |
| --- | --- | --- | --- | --- | --- |
| **Low-risk youth as the comparison group** | | | | | |
| Lampinen  2006 | Self-reported MA use  18.5% used MA within previous month | Other drug use | Use of alcohol, marijuana, tobacco, mushrooms, ecstasy, cocaine, LSD, ketamine, GHB, heroin, or peyote along with MA | Sexual behavior | MA use is highly correlated with other drug use; preventative programs should not be MA specific but address drug use in general |
| Oetting  2000 | Self-reported MA use  1.9-2.5% used MA in the previous month | Sexual behavior |  | Ethnicity | Males, American Indians and Hispanics are more likely to use MA |
| Sattah  2002 | Self-reported MA use  35% had positive MA urine tests | Sexual behavior |  | Age  Sexual history  Other drug use  Living with family  Parents’ marital status  Depression | Male sex, other substance use, sexual activity, and absence of family are all correlated with MA use |
| Yen  2006 | Positive MA  urine test  40% used MA >4 times/month | Psychiatric  co-morbidity | Diagnosis of conduct disorder, ADHD, adjustment disorder, anxiety disorder, depressive disorder, oppositional defiant disorder, bipolar I disorder, eating disorder, or any psychiatric disorder | Attitudes towards MA  MA knowledge  Family characteristics  Other drug use  Peer factors | Conduct disorder, ADHD, adjustment disorder, favourable attitude toward MA, poor MA knowledge, disrupted parenting, peer factors, and other drug use are all correlated with MA use |
| Yen  2004 | Positive MA  urine test  Mean monthly MA use of 14.3 (SD 20.0) | Risky sexual behavior | Age at first time of intercourse, total number of sexual partners, frequency of intercourse in the last month, unprotected sex, unplanned sex under the influence of MA or alcohol, sex with a partner under the influence of MA or alcohol, sex immediately after MA use, opinion of aphrodisiac qualities of MA | Age  Education  Age at initial MA use | There is a strong relationship between MA use and risky sexual behavior; risk reduction programs are strongly needed |
| **High-risk youth as the comparison group** | | | | | |
| Kim  2002 | Self-reported MA use | Sexual behavior |  |  | Females are more likely than boys to become MA dependent |
| Miura  2006 | History of MA use determined upon physical examination | History of violence | Family history of crime, alcohol abuse, drug use, or child abuse and personal history of violence | Age  Psychiatric treatment  Number of admissions | MA users are less violent than non-MA users from a juvenile classification home. Female sex, increased age, disturbed family history, and psychiatric treatment are related to MA use |
| Palmer  2005 | Diagnosed with MA dependence | Personality characteristics | Minnesota Multiphasic Personality Inventories-Adolescent was used to look for elements of personality disorders | Ethnicity | Personality profiles of MA users do not significantly differ from hallucinogen or cannabis users |
| Rawson  2005 | Self-reported lifetime MA use | Sexual behavior |  | Age  Ethnicity  Family history of drug use | Female sex, increased age, and psychosocial dysfunction are  correlated with MA use |
| Shillington  2005 | Self-reported MA use | Parental monitoring | How often guardians knew where their adolescents were, who they were with, and what they were doing (never, sometimes, or always) | Alcohol use  Drug use | Adolescents reporting low levels of parental monitoring are significantly more likely to use MA |
| Shillington  2003 | Self-reported MA use  MA use was primary drug for entering treatment | Sexual behavior |  | Ethnicity | Females are more likely to use MA |
| Uchida  1995 | 89% used MA 20 times | Disrupted family history | Family history of an alcoholic parent or child abuse | Sex behavior  Suicidal thoughts Suicide attempts | MA use and a history of child abuse, an alcoholic parent, suicidal thoughts, and suicidal thoughts are correlated |
